# Extended Emitter Target Tracking Using GM-PHD Filter

**DOI:** 10.1371/journal.pone.0114317

**Published:** 2014-12-09

**Authors:** Youqing Zhu, Shilin Zhou, Gui Gao, Huanxin Zou, Lin Lei

**Affiliations:** College of Electronic Science and Engineering, National University of Defense Technology, Changsha, Hunan, China; Southwest University, China

## Abstract

If equipped with several radar emitters, a target will produce more than one measurement per time step and is denoted as an extended target. However, due to the requirement of all possible measurement set partitions, the exact probability hypothesis density filter for extended target tracking is computationally intractable. To reduce the computational burden, a fast partitioning algorithm based on hierarchy clustering is proposed in this paper. It combines the two most similar cells to obtain new partitions step by step. The pseudo-likelihoods in the Gaussian-mixture probability hypothesis density filter can then be computed iteratively. Furthermore, considering the additional measurement information from the emitter target, the signal feature is also used in partitioning the measurement set to improve the tracking performance. The simulation results show that the proposed method can perform better with lower computational complexity in scenarios with different clutter densities.

## Introduction

As a means of avoiding the complicated problem of data association, the probability hypothesis density (PHD) filter [1] has received considerable attention in multi-target tracking [2]–[9]. Like traditional tracking algorithms, the standard PHD filter assumes that each target produces at most one measurement per time step. However, with the application of high-resolution sensors, one object (e.g., large airplane and ship) may yield several measurements at each time step and is then denoted as an extended target. In a passive tracking system, the sensor (e.g., electronic support measure sensor) usually locates the target by detecting the electromagnetic wave emitted by the target's radar. When there are several radars equipped on the target, the sensor will receive more than one measurement from the target at a given time step. Thus, emitter target tracking relates to the problem of extended target tracking, which has been a research hotspot in recent years. Gilholm *et al.* suggested a non-homogeneous Poisson point process measurement model in extended target and group tracking [10]. On that basis, an improved PHD filter for handling extended targets was proposed by Mahler [11]. Then, Granstrom *et al.* presented a Gaussian-mixture implementation of the PHD filter for extended target tracking in the linear Gaussian system [12]. Similarly, a Gaussian-mixture implementation of the CPHD filter is also presented in reference [13], where experiments using real data from a laser sensor show that the extended target CPHD filter exhibits better performance than the PHD filter in estimating the number of targets.

The purpose of a measurement set partition is to cluster the measurements from the same target into one cell, which is used to update the intensity. It is an important part of the extended target PHD filter (ET-PHD). The validity of the measurement set partition directly affects the tracking performance; however, the computational complexity increases sharply as the number of possible partitions increases. A simple solution is to use K-means clustering to generate partitions according to different values of K. In view of its sensitivity to the initialization of the algorithm, an improved version called K-means++ [14] can be chosen to overcome this problem. Granstrom *et al.* proposed a distance partitioning method using a set of distance thresholds to obtain the cells [12]. Furthermore, a sub-partitioning approach is added to handle the close-spaced targets [15]. After distance partitioning, the method takes advantage of the maximum likelihood (ML) algorithm to estimate the number of targets in each cell. If the number is larger than one, the cell will be split into smaller cells. In their recent work [16], Zhang and Ji presented a novel fast partitioning algorithm based on the Neural Network (NN), where the Fuzzy ART model is used with different vigilance values to partition the measurement set. However, certain shortcomings may still exist in the above methods, such as high computational complexity, inaccurate partitions and the difficulty of setting parameters. Therefore, a fast, simple and valid partitioning algorithm for the ET- PHD filter is necessary. In this paper, a new partitioning algorithm based on hierarchy clustering is proposed. It iteratively computes the pseudo-likelihoods to achieve fast tracking of extended targets through the use of the neighboring partitions.

Additionally, in a passive tracking system, certain signal features of the emitter, such as radio frequency (RF), pulse repetition interval (PRI) and pulse width (PW), can be received in addition to the location information. They represent the characteristics of the emitter and play an important role in the classification and recognition of the emitter [17]–[19]. As a result, this paper tries to incorporate the signal features into the ET-PHD filter to improve tracking performance in cluttered environment.

The remainder of this paper is organized as follows. The Background section mainly reviews the Gaussian-mixture implementation of the ET-PHD (ET-GM-PHD) filter. The measurement set partition based on hierarchy clustering and the modified ET-GM-PHD filter incorporating the signal features of the emitter are detailed in the Method section. Then, in Results and Discussion, the simulation results are analyzed to validate the proposed method. Conclusions are presented in the final section.

## Background

In a linear Gaussian system, the GM-PHD filter can easily estimate the target statements and the number of targets. Without considering the spawned target, the Gaussian-mixture implementation of the ET-PHD filter can be given by the following three steps [3], [12], [15] which present a closed form solution to the PHD recursion.

### Prediction

Assume that the posterior intensity at time *k*–1 is a Gaussian-mixture form
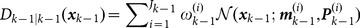
(1)where 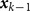 represents the target statement at time *k*–1, 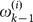 is the weight of the *i^th^* component, and 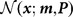 denotes a Gaussian density with mean ***m*** and covariance ***P***. Then, the predicted intensity at time *k* is given by
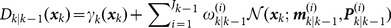
(2)where 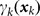 is the birth intensity
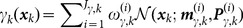
(3a)

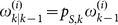
(3b)

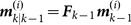
(3c)

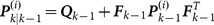
(3d)where 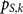 represents the survival probability, 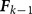 is the transition matrix of the system, and 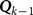 is the process noise covariance.

### Update

Because the birth intensity is also a Gaussian-mixture form, the predicted intensity can be expressed as
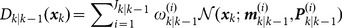
(4)where 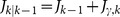. Then, the posterior intensity 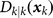 can be updated by
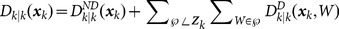
(5)


The notation 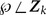 means that 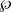 partitions the measurement set 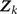 into cells *W*. Under assumption 8 in reference [15], 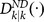 handling the no detection cases can be given by
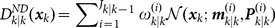
(6)where
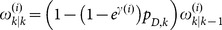
(7a)

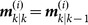
(7b)

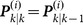
(7c)where 
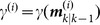
 is the approximation of the expected number of generated measurements 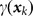, and 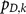 denotes the detection probability. Similarly, 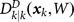 handling the detected target cases is given by
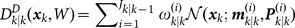
(8)and the weight is
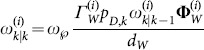
(9a)

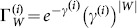
(9b)

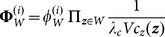
(9c)where 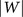 is the number of elements in *W*, 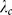 is the average clutter density, *V* is the surveillance region, and 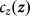 is the probability density of the spatial distribution of clutters. The coefficient 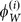 can be given by
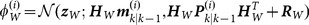
(10a)

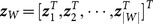
(10b)

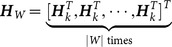
(10c)

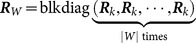
(10d)where 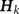 is the measurement matrix of the sensor, and 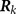 is the observation noise covariance. The weight of partition 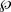 is
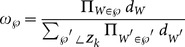
(11a)

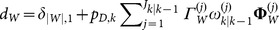
(11b)where 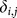 is the Kronecker delta. The means and covariances of the Gaussian-mixture 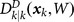 are
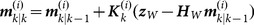
(12a)

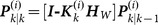
(12b)

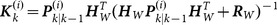
(12c)


From the above equations, we can see that these coefficients need to be calculated over again for each partition, which will cost much time in the recursion.

### Target statement estimation

After the update, the pruning and merging procedures are always used to reduce the number of Gaussian components [3]. The new intensity can be rewritten as
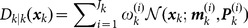
(13)


Then, the means of the Gaussians with greater weights can be selected as the estimates of the target statements. For example,
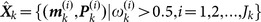
(14a)

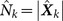
(14b)where 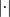 is the cardinality of the set, and the number of targets 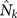 can also be estimated.

## Methods

For the purpose of reducing the computation in the update step and having a good performance, the partitioning algorithm for the measurement set based on the hierarchy clustering is presented at first in this section. Secondly, we describe how to make use of the partitioning algorithm to iteratively calculate the coefficients in the ET-GM-PHD filter. Then, the signal feature of extended emitter target is used to improve the tracking performance. Finally, the computational complexity of the proposed method is analyzed.

### Partitioning the measurement set

The number of all possible partitions in the ET-PHD filter will increase sharply with the number of measurements. Thus, this filter is computationally intractable for real application. Therefore, a partitioning algorithm with a set of parameters is usually used to approximate the measurement set partitions. The hierarchical agglomerative clustering algorithm is a commonly used method in data analysis that continuously combines the clusters to partition the data set. In view of its simplicity and low computational complexity, the single-link (SL) hierarchical [20], [21] method is chosen to partition the measurement set, which is described as follows:

Input: the measurement set 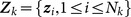, where 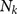 is the number of measurements.

Step 1. (Initialization)

For each 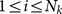, let 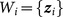. Let 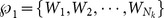 and 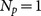. Define a distance matrix 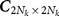 where 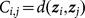 for 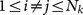. The values of other elements in 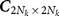 are set to be Inf (positive infinity).

Step 2. (Loop)

While 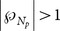


Find 
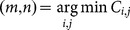
. Let 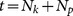 and 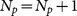. Then, we can obtain a new partition 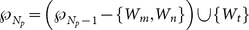, where 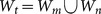. Update the distance matrix. Calculate the new distance 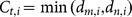 for each 
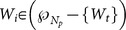
. Let 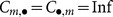 and 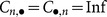.

End

Output: the measurement set partitions 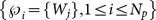, where 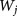 is the cell, and 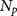 is the number of partitions.

It can be seen that the proposed algorithm only combines the two cells with the shortest distance from the last partition to obtain a new partition at each time point. Consequently, the cells in the two neighboring partitions 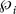 and 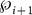 are almost the same. This characteristic can save substantial time in computing the pseudo-likelihoods in the update step of the PHD filter.

### ET-GM-PHD filter based on hierarchy clustering partitions

As shown in Background, the measurement set partition only affects the Gaussian-mixture 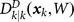. Supposing that there are 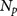 partitions, the updated posterior intensity can be changed as follows in form:
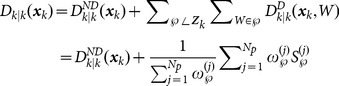
(15)where 
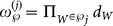
(16a)

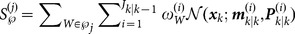
(16b)

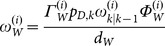
(16c)


Then, we assume that 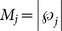 is the number of cells in the 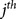 partition denoted as 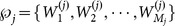, and let 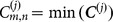. Using the partitioning algorithm described in the above, the new partition can be denoted as 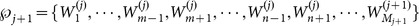, where 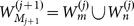. According to Equation (16a–c), we have
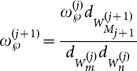
(17)

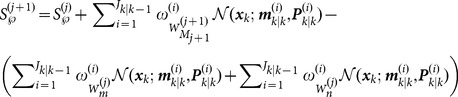
(18)


Thus, 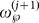 and 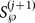 can be calculated iteratively, and the additional computation only relates to the new cell 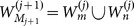. The number of new Gaussian components generated by the new partition 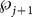 is only 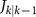. The other Gaussian components in 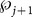 are the same as those in 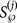, and the weights can be accumulated directly in the update step. As a result, the ET-GM-PHD filter based on hierarchy clustering can effectively reduce the number of new Gaussian components, as well as the computation caused by the different partitions, through the iterative process.

### Combining extended emitter target tracking with signal features

To improve the performance of the extended emitter target tracking, we try to incorporate the signal features into the ET-GM-PHD filter. Suppose that the augmented emitter target statement consists of the kinetic information 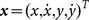 and the signal feature information 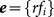. (For convenience, we assume that the signal feature only contains RF information.) It should be noted that there may be more than one RF, which means that one emitter target may generate several measurements per time step. The augmented statement of the extended emitter target is denoted as
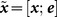
(19)


Assuming that the signal feature is independent of the target motion statement and not time-varying, the posterior intensity at time 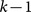 can be denoted as
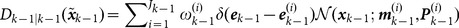
(20)where 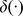 is the Dirac delta. The birth intensity can also be denoted as
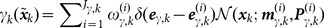
(21)


And the predicted intensity at time 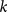 can be given by
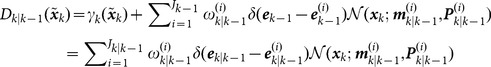
(22)


Suppose that the measurement is
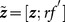
(23)where 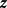 is the measurement information of the target location, and 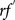 is the measurement information of RF.

Then, the signal feature can be integrated into the measurement set partition. The distance between two arbitrary measurements 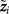 and 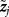 can be defined as
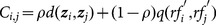
(24)where 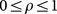 is a free parameter, 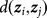 represents the location distance, and 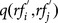 represents the distance of the signal feature, which is related to the emitter target. For example, assuming that 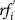 is very different from 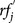, 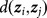 will be small when they come from the same target. To partition them into the same cell, 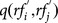 must be small as well. However, when they are from different targets, 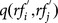 should be large to avoid putting them in the same cell. Thus, the distance 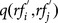 is defined as
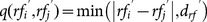
(25a)

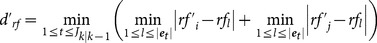
(25b)


Intuitively, it can be interpreted as follows:

Suppose that there are 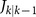 middle nodes in the network. From the start node *i* to the end node *j*, one can either select a direct route 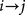 or an indirect route 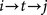 through a middle node *t*. Then, 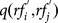 represents the shortest walking distance between nodes *i* and *j*. When given a parameter 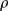, the measurement set can be partitioned by the hierarchy method described in the first part of this section.

### Computational complexity analysis

Since different parameters will lead to different number of measurement set partitions for the algorithm, in order to facilitate the analysis, we assume that all the algorithms have the same number of the partitions. To obtain a new partition 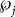, the complexity of Distance Partitioning [12] is approximated as 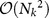
(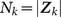 is the number of the measurements), the complexity of K-means++ Partitioning [15] is approximated as 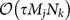
(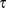 is the iterative time, and 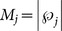 is the number of the cells in the partition), and the complexity of Fuzzy ART Partitioning [16] is 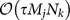 as well. Whereas, the complexity of our partitioning algorithm is only 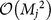 because the new partition 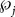 can be obtained directly from the last partition 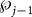.

When given a new partition, in most cases, the coefficients need to be calculated over again in the update step because the new partition is always very different from the acquired partitions. The complexity of other existing algorithms is approximated as 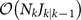
(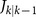 is the number of Gaussian components in the predicted intensity 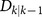). However, due to the similarity between the two neighboring partitions 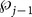 and 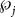 the complexity of the proposed algorithm is approximated as 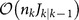
(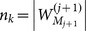 is the number of measurements in the new cell, and 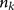 is not larger than 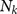 obviously). In summary, our method has a lower computational complexity than other existing algorithms.

## Results and Discussion

The elapsed time and the optimal subpattern assignment (OSPA) metric [22], which can measure errors in both location and target number, are adopted for performance evaluation. The algorithm with smaller OSPA distance will exhibit better performance. Details can be found in reference [22].

### Materials

Assuming that the sensors and targets are in a uniform Cartesian coordinates system, the target statement vector is denoted as 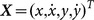, which contains the positions and velocities of the X-axis and Y-axis. The motion model of the target is constant velocity (CV),
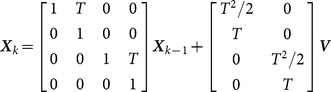
(26)where 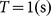 is the sampling interval, and the process noise 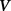 is a sequence of zero-mean Gaussian noise with covariance matrix 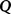


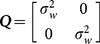
(27)where 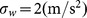 in the experiments. The measurement is given by
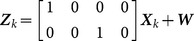
(28)where the measurement noise 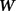 is also a sequence of zero-mean Gaussian noise whose covariance matrix is
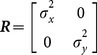
(29)where *σ_x_* = *σ_y_* = 10(m) in the experiments.

The probability of target detection is *p_D,k_* = 0.98, and the length of the time step is *K* = 50(s). The location of clutter with Poisson RFS is uniform over the surveillance region *V* = [0, 1200]×[0, 1200](m^2^). The radio frequency of the clutter is uniform in the range of [0, 5000](MHz). In the ET-GM-PHD filter, the probability of survival is *p_S,k_* = 0.95, the maximum number of Gaussian component is *J*_max_ = 100, and the pruning and merging thresholds are *T_prune_* = 10^−5^ and *U_merge_* = 4 respectively. The parameters of the OSPA metric are set to *p* = 2 and *c* = 100.

**Figure 1:**
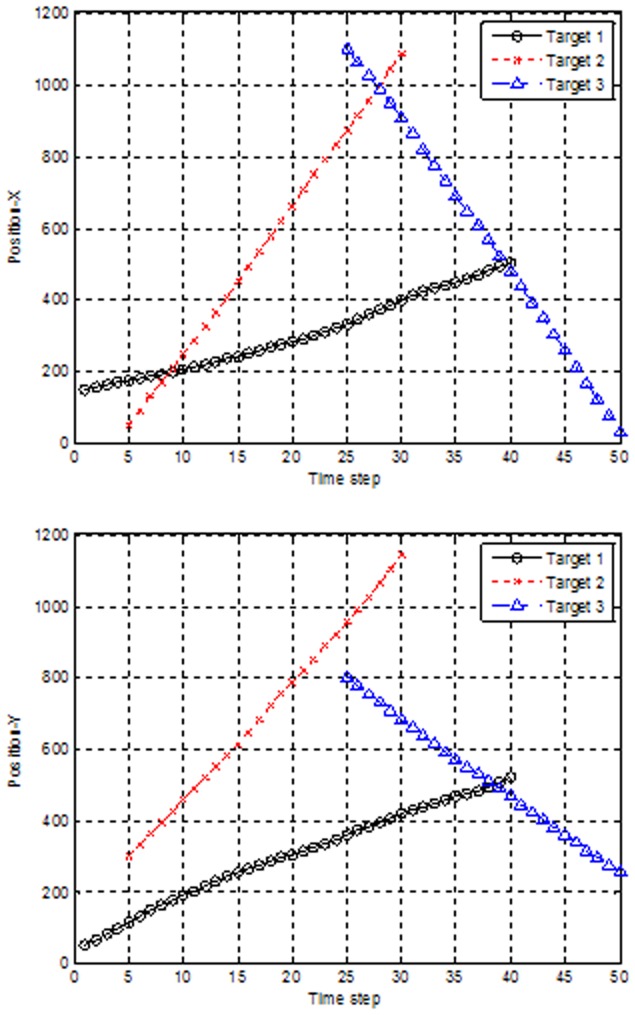
Target trajectories.

We conducted two series of experiments in this section. First, the proposed partitioning algorithm based on hierarchy clustering is compared with three other methods for the ET-GM-PHD filter. Second, the effect of the signal features on improving the tracking performance is validated. Monte Carlo experiments are repeated 200 times for each case. The simulations are implemented in MATLAB on an Intel Core i5-2320 3.00 GHz processor with 3.5 GB RAM. There are 3 targets moving in the surveillance region, as illustrated in Fig. 1.

### Experiment 1

Without considering the signal features of the emitter target, the first series of experiments mainly compares the performance of the proposed method with other partitioning algorithms based on K-means++ [15], Distance Partitioning [12], and Fuzzy ART [16]. The number of clusters is 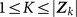 in K-means++; the probability threshold is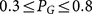 in Distance Partitioning; and in Fuzzy ART, the vigilance threshold is set to be 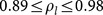 with 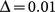. The birth intensity is given by
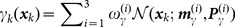
(30)where 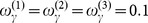
(31a)

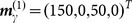
(31b)

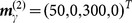
(31c)

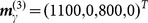
(31d)

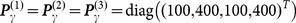
(31e)


**Figure 2:**
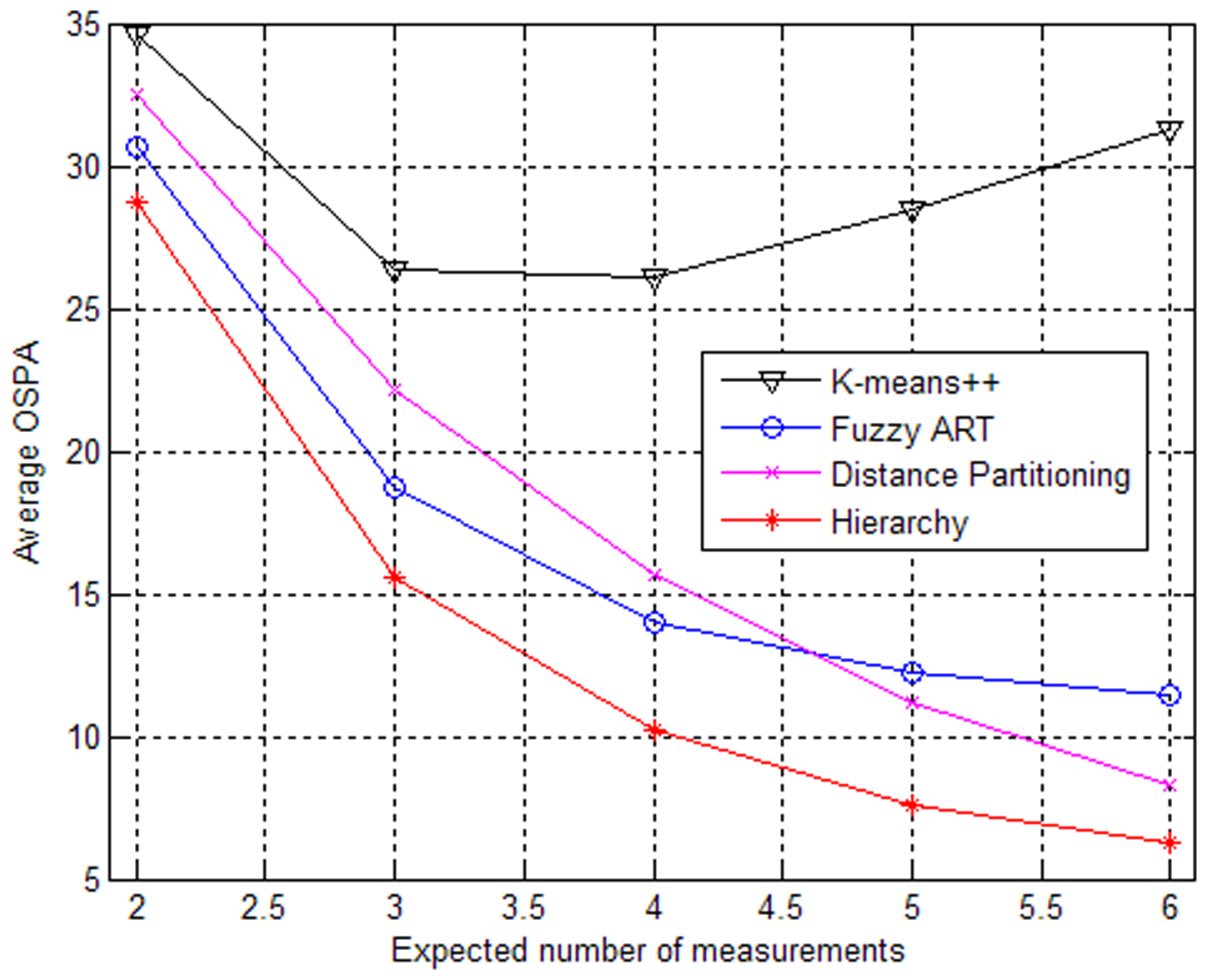
Performances of partitioning algorithms versus expected number of measurements.

**Figure 3:**
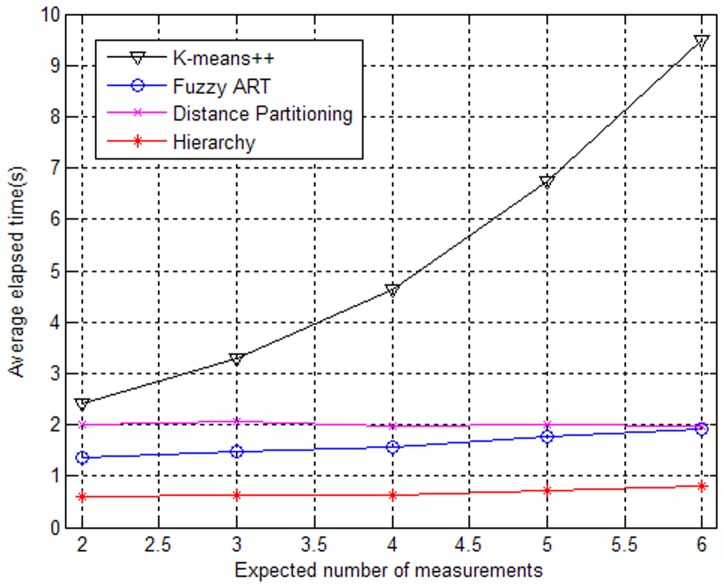
Elapsed times of partitioning algorithms versus expected number of measurements.

Let 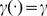 be a constant in the ET-GM-PHD filter. With clutter density 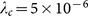 (i.e., seven clutter returns per scan over the region), the impacts of different expected numbers of generated measurements on the algorithms are shown in Fig. 2 and Fig. 3.

From Fig. 2, it can be seen that the partitioning algorithms based on Fuzzy ART, Distance Partitioning and hierarchy clustering perform better as the expected number increases, which is because the clutter will have less negative effect on the tracking performance when the number of the measurements from targets increases, and the clutter density is invariable. Similarly, a high signal to noise ratio (SNR) will lead to good performance. However, the partition based on K-means++ does not perform well. The main reason is that K-means++ often fails to obtain the correct partitions due to the existence of counter-intuitive local optima for the implicit cost function [16]. In addition, as shown in Fig. 3, we can see that the elapsed times increase only slightly with the expected number increasing except for the partition based on K-means++. The proposed partitioning algorithm based on hierarchy clustering has a clear advantage in both tracking performance and elapsed time.

**Figure 4:**
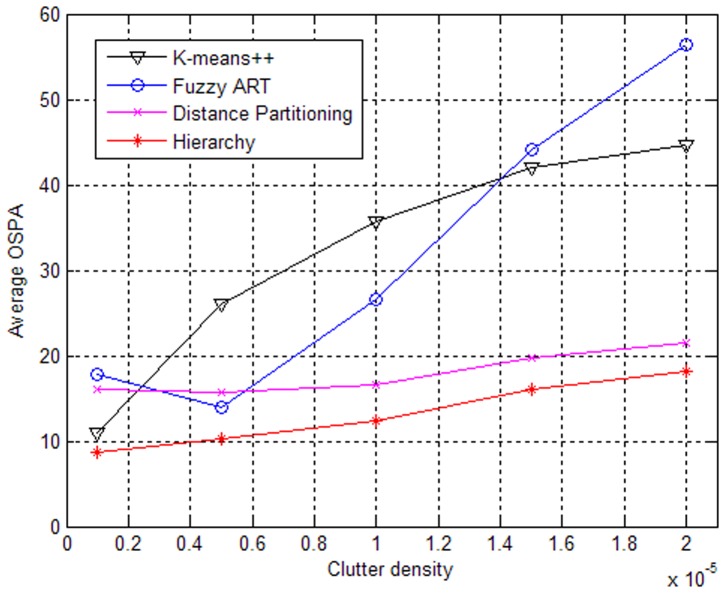
Performances of partitioning algorithms versus clutter density.

**Figure 5:**
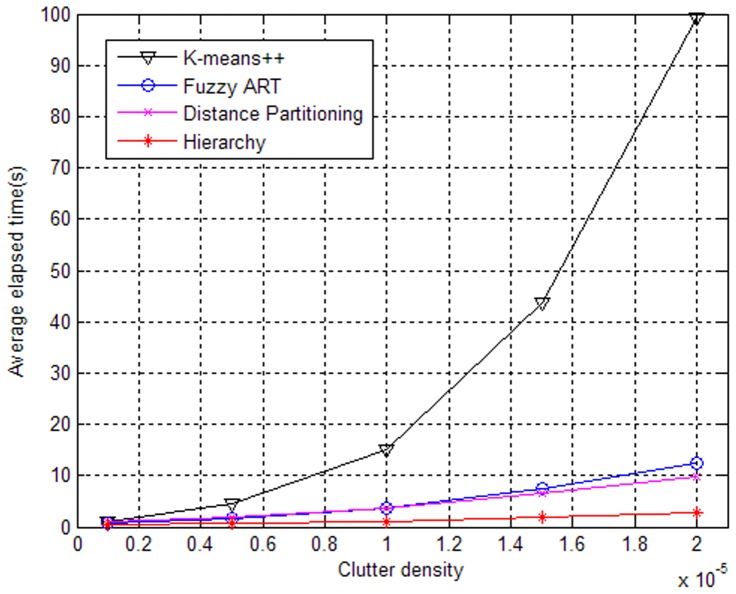
Elapsed times of partitioning algorithms versus clutter density.

To further validate our approach, another experiment is performed in scenarios with different clutter densities. The results are illustrated in Fig. 4 and Fig. 5 when the expected number of generated measurements is γ = 4. Fig. 4 shows that the performances will drop as a whole when the clutter density becomes large. However, the performance of the partition based on Fuzzy ART changes greatly. One reason may be that the vigilance thresholds are not adapted to our simulation, which causes overestimation of the target number and leads to a large average OSPA distance. From Fig. 5, it can be seen that the elapsed time of the partition based on K-means++ increases sharply with increasing clutter density. Overall, the ET-GM-PHD filter with the proposed partitioning algorithm can handle extended target tracking better in cluttered environment.

### Experiment 2

In the second experiment, we want to know whether the PHD filter combined with the signal feature exhibits better performance for extended emitter target tracking. For simplicity, we assume that the signal of emitter (RF) is not time-varying and only has some jitter:
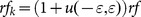
(32)where 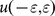 is a uniform distribution in the range of 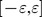 (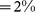 in the experiments). The measurement is given by
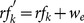
(33)where 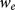 is zero-mean Gaussian white noise with standard deviation 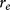. The birth intensity is given by
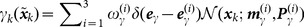
(34)where 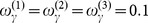
(35a)

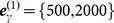
(35b)

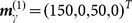
(35c)

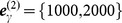
(35d)

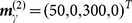
(35e)

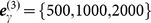
(35f)

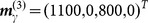
(35g)

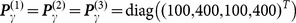
(35h)


**Figure 6:**
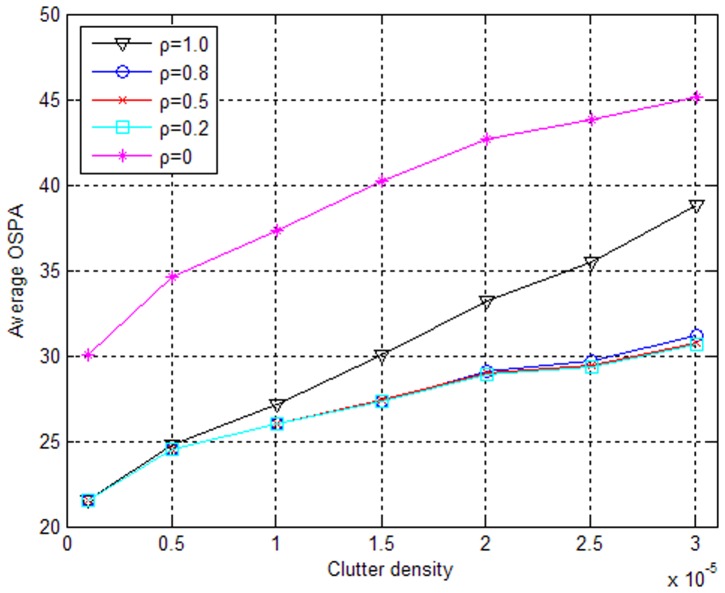
Performance comparison in combination with RF when *r*_e_ = 10(MHz).

**Figure 7:**
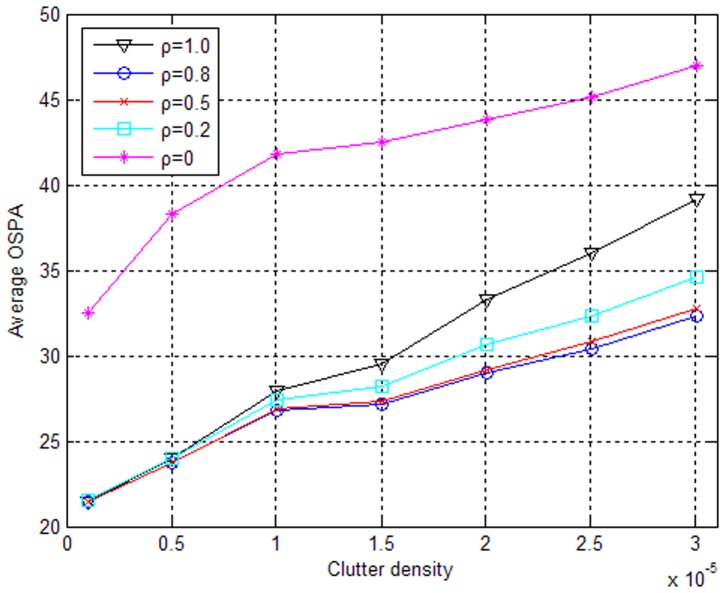
Performance comparison in combination with RF when *r*_e_ = 100(MHz).

Let 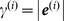 in the ET-GM-PHD filter, meaning that the expected number of measurements generated by the emitter target is equal to the number of equipped radars and is reasonable in real application. Incorporating the signal feature (RF) into the measurement set partition, the tracking performances with different values of the free parameter *ρ* in Equation (24) are illustrated in Fig. 6 and Fig. 7. From these figures, we can see that neither considering only the location information (*ρ* = 1) nor considering only the RF information (*ρ* = 0) exhibits good performance. This result is because that high clutter density will have a bad effect on the partitioning algorithm based on the location information, but there is overlapping of RF between different targets so that a measurement set partition based solely on the RF information will not be correct. In contrast, considering both the location and the RF information (0<*ρ*<1) will improve the performance of the modified ET-GM-PHD filter to varying degrees, effectively validating the auxiliary function of the signal feature information. In addition, Fig. 6 shows that there are few differences in the improvements of the algorithm performance with different values of *ρ* (*ρ* = 0.2,*ρ* = 0.5,*ρ* = 0.8) when the measurement noise of location (*σ_x_* = *σ_y_* = 10) and the measurement noise of RF (*r_e_* = 10) are small. However, when the measurement noise of RF (*r_e_* = 100) is large, considering the relatively accurate location information (*ρ* = 0.8) more heavily is more conducive to improving the tracking performance.

## Conclusions

To address the problem of measurement set partitioning in the ET-PHD filter, this paper proposes a fast partition method based on hierarchy clustering. It can iteratively compute pseudo-likelihoods according to the neighboring partitions. In addition, the signal feature is incorporated into the modified ET-GM-PHD filter for extended emitter target tracking. The simulation results show that compared with other partitioning algorithms, the proposed partition algorithm based on hierarchy clustering not only exhibits better performance but greatly reduces the computational complexity. Combining the PHD filter with the signal features can effectively improve tracking performance in the simulated scenarios.

Because the CPHD filter has an advantage in the estimation of the target number, another future work is to extend the proposed approaches to the ET-CPHD filter.
